# Salvage Treatment for a Periprosthetic Humeral Nonunion: A Case Report

**DOI:** 10.7759/cureus.60491

**Published:** 2024-05-17

**Authors:** Kassidy Webber, Blake E Delgadillo, Samuel Shepard, Ania Bartholomew, Adam Dann

**Affiliations:** 1 Orthopaedic Surgery, Lake Erie College of Osteopathic Medicine, Bradenton, USA; 2 Orthopaedic Surgery, Kettering Health Network, Dayton, USA; 3 Orthopaedic Surgery, Orthopaedic Associates of Dayton, Dayton, USA

**Keywords:** periprosthetic fracture, reverse total shoulder, salvage, total humeral replacement, allograft, periprosthetic

## Abstract

The patient, a 69-year-old female, presented one year after receiving a total elbow arthroplasty with a nonunion periprosthetic fracture of the humerus. Due to the patient's severe osteoarthritis of the ipsilateral shoulder and significant humeral deformity, a procedure linking the total elbow arthroplasty to the reverse shoulder implant via a cemented allograft-composite linkage sleeve was performed. Previous literature suggests upper extremity salvage surgery using large-scale allografts is successful in treating large tumor or infection-derived defects, though data is lacking as to whether this treatment is effective in periprosthetic fractures in patients with significant comorbidities. This patient's success in the postoperative year supports the use of allograft-composite reconstruction followed by linkage to a reverse shoulder implant as a salvage treatment for periprosthetic fractures under certain conditions, such as multiple adjacent implants, bone deformity, and severe osteoarthritis.

## Introduction

Salvage surgery is an operation that utilizes allograft or other last-resort measures for severe insults that other management has failed to repair [1,2]. Salvage-type surgeries are commonly utilized for large defects secondary to malignancy, infection, or, less commonly, fracture [2-5]. Although there is controversy over standard fixation methods, in the upper extremity, common techniques include soft tissue reconstruction, revascularization, and rigid stabilization [1]. Children, adolescents, and young adults are among those who typically undergo salvage surgery due to bone pathology, although metastatic bone processes are an exception to this norm [2]. Monahan et al. demonstrated the use of a total elbow arthroplasty (TEA) with femoral strut allograft in a patient with a prosthetic joint infection, distal humeral bone loss, and incomplete union of a periprosthetic humeral shaft fracture [5]. This study postulated the use of a staged revision with strut allograft combined with a TEA permitted increased fracture support, implant stability, earlier return of function, and increased strength [5]. Chin and Lambert discuss how not all TEAs are successful, which is due to a variety of causes, including systemic disease, regional muscular or neurologic issues, articular dysfunction, device failure, and, most challengingly, infection [6]. Annually, the total number of revision TEAs remains low, but this procedure accounts for a high proportion of all TEAs. These authors discuss the importance of targeted post-op aftercare, which highly depends on the stability of the implant and bone, as well as the reconstructed extensor mechanism. Patients are typically advised against weight-bearing in the operative extremity, but they are encouraged to supinate and flex the elbow against gravity, given the skin closure remains intact. After about three months, implant and bone stability are usually achieved, which allows patients to begin weight-bearing along with load-sharing using long lever arm movements. In general, heavy weight-bearing and high-impact activities should be avoided. The rehabilitation plan should be tailored to each individual's goals and needs. When treating osteosarcoma of the humerus, one of the most common sites of this malignancy, total humeral replacement (THR), is employed in cases with severe tumor burden to effectively eliminate the cancer [7]. In this procedure, the anatomy of the glenohumeral joint is reversed, and the prosthesis is attached distally via a TEA. Tran et al. explored the use of THR in a case of osteosarcoma that employed lighter polymer materials which resulted in a superior functional outcome during the rehabilitation phase compared to previously used heavyweight titanium alloys [7]. 

The case presented in this manuscript demonstrates a similar procedure to THR with concomitant use of cortical allograft for a periprosthetic nonunion fracture of the distal humerus complicated by severe osteoporosis. This procedure employed allograft-composite reconstruction utilizing a cadaveric bone graft and a prosthesis with cement to address the complex defect.

## Case presentation

**Figure 1:**
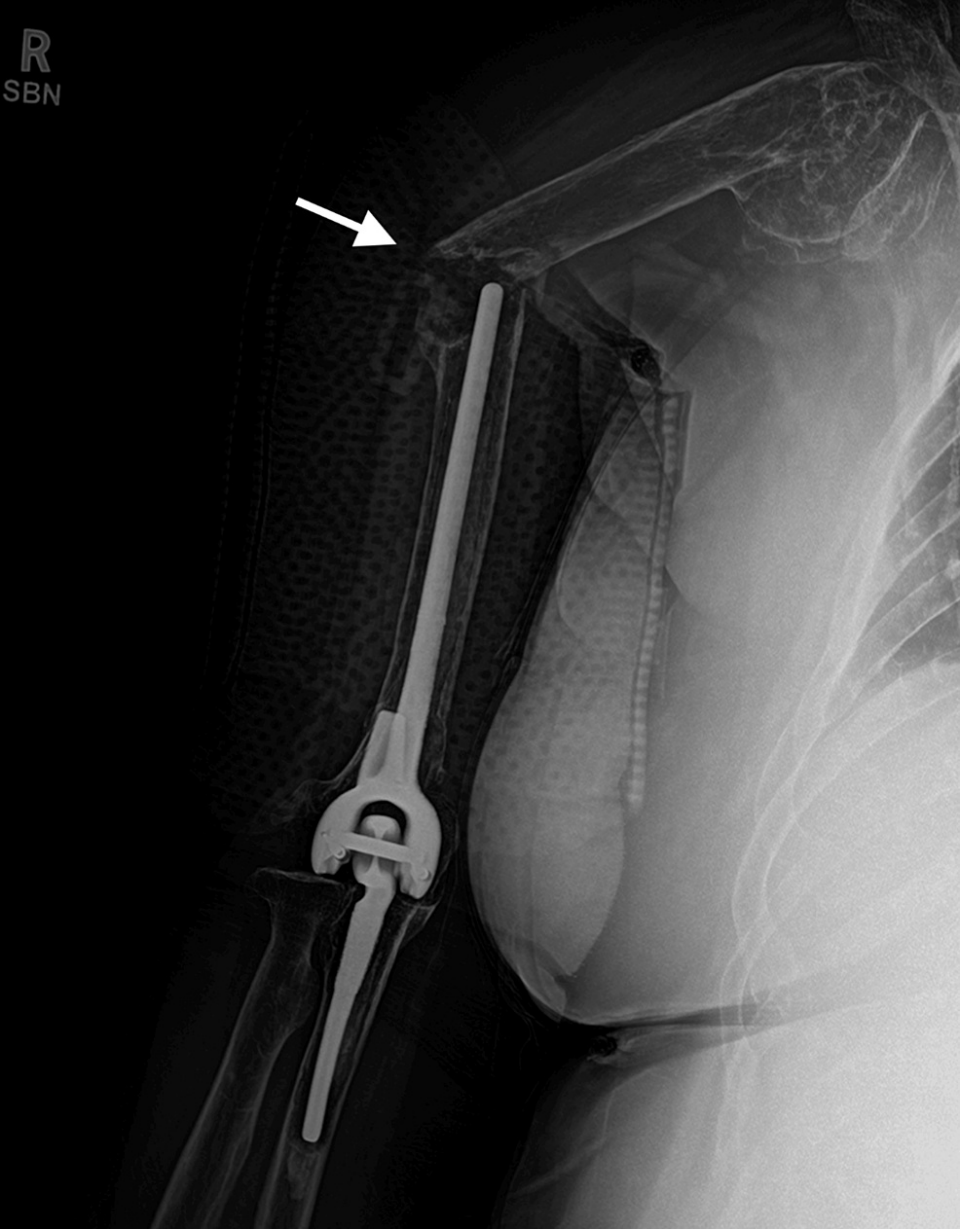
Preoperative radiograph of periprosthetic humerus fracture. Arrow designates periprosthetic humerus fracture.

The patient, a 69-year-old female, presented with complaints of right arm pain and decreased range of motion three days after a traumatic fall in February 2022. The patient has an extensive medical history, including a proximal humerus malunion, generalized osteoarthritis, osteoporosis, chronic pain, uterine cancer, analgesic overdose, seizure, and migraines. Past surgical history includes thoracic spine surgery, hysterectomy, and open reduction and internal fixation (ORIF) of the left elbow and bilateral knees. Additionally, she had a right TEA with ulnar nerve neurolysis secondary to a comminuted intra-articular distal humerus fracture in January 2019. Radiologic workup revealed a mid-shaft humerus fracture immediately proximal to an elbow implant as shown in Figure 1.

**Figure 2:**
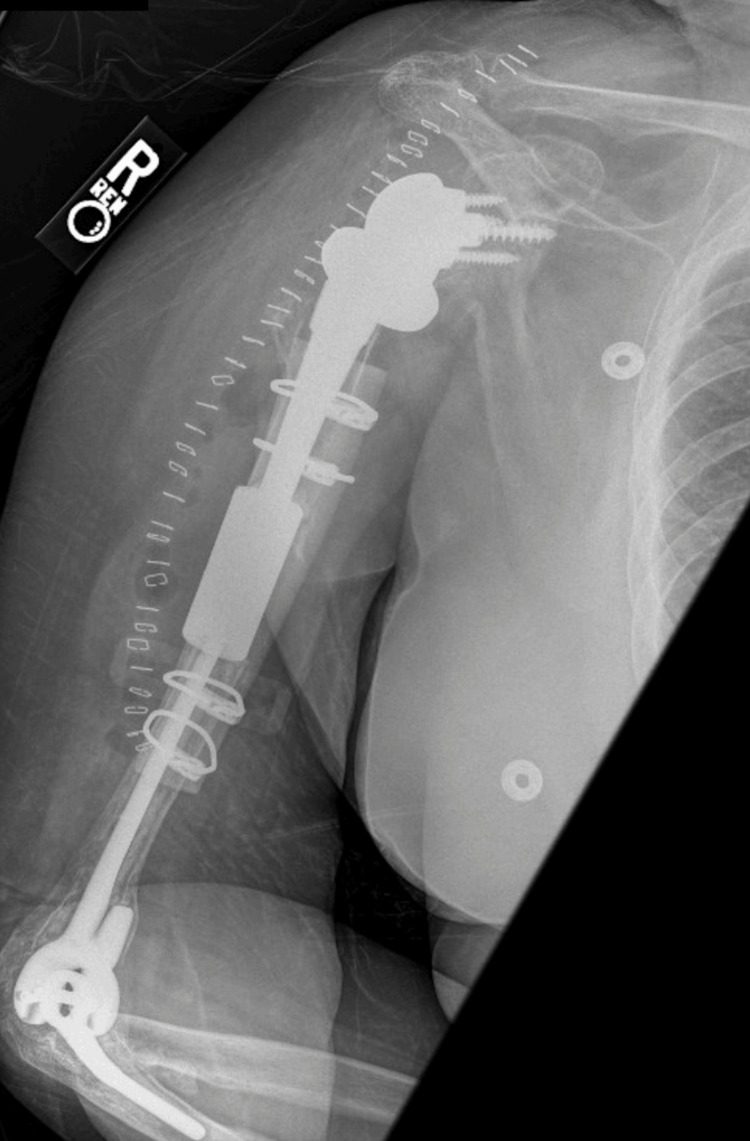
POD0 lateral radiograph of the right humerus with allograft repair and RTSA. POD: postoperative day; RTSA: reverse total shoulder arthroplasty

**Figure 3:**
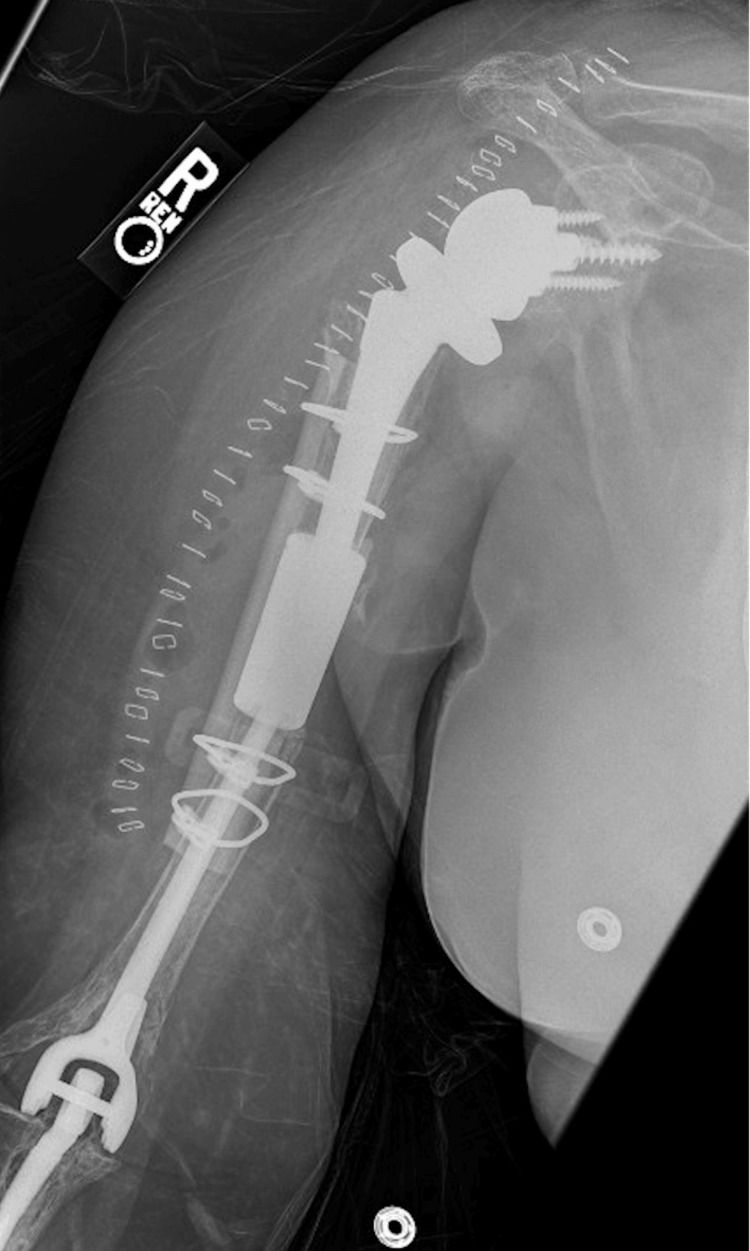
POD0 AP radiograph of the right humerus with allograft repair and RTSA. POD: postoperative day; AP: anteroposterior; RTSA: reverse total shoulder arthroplasty

The patient elected to pursue conservative management at the time of presentation, and the pain improved over the following weeks. In February 2023, she presented for follow-up with worsening pain. Plain radiograph imaging revealed nonunion at the original periprosthetic fracture site. The patient consented to surgical intervention to remedy the osteoarthritis at the shoulder joint, address the nonunion, and reinforce the existing bone structure at stress risers. Later that month, after careful consideration, the operative team and patient decided to pursue an allograft-composite reconstruction with linkage to a reverse total shoulder arthroplasty (RTSA) for a peri-TEA nonunion fracture of the distal humerus. The patient was brought to the operative suite, and anesthesia was induced in a standard fashion. A deltopectoral incision was made and extended over the anterior aspect of the arm. The nonunion site was initially addressed by removing the hypertrophic callus and mobilizing the fracture site. The proximal humerus was then exposed, and a freehand cut of the humeral neck removed the head and a portion of the greater and lesser tuberosities. Next, the nonunion site was addressed by measuring the area in which the linkage sleeve would be placed and performing an osteotomy of the mid-distal humerus to allow the stem of the TEA to insert into the linkage sleeve. Sequential broaching was performed up to a size 9 mini-broach. The osteotomy of the proximal segment was performed so that the stem of the humeral shoulder implant would insert into the sleeve linkage. After clearing the soft tissue and protecting the radial nerve, four cerclage cables were placed, two proximal and two distal to the nonunion site. Cement was applied circumferentially around the proximal aspect of the elbow's humeral component and within the linkage sleeve which was then impacted onto the distal component. The shoulder's humeral component was then impacted on the proximal humerus, and the tip of the stem was seated into the custom linkage and reinforced with cement. After the cement was cured and ridged fixation was achieved, the cortical strut allograft was placed along the anterior humerus, and the tension of the cerclage cables was readjusted to appropriately bridge the linkage sleeve. Once the linkage sleeve was secure, a customary RTSA was performed. Standard postoperative X-rays were obtained as shown in Figure 2 and Figure 3. The postoperative hospital course was uncomplicated, and the patient was discharged three days later.

**Figure 4:**
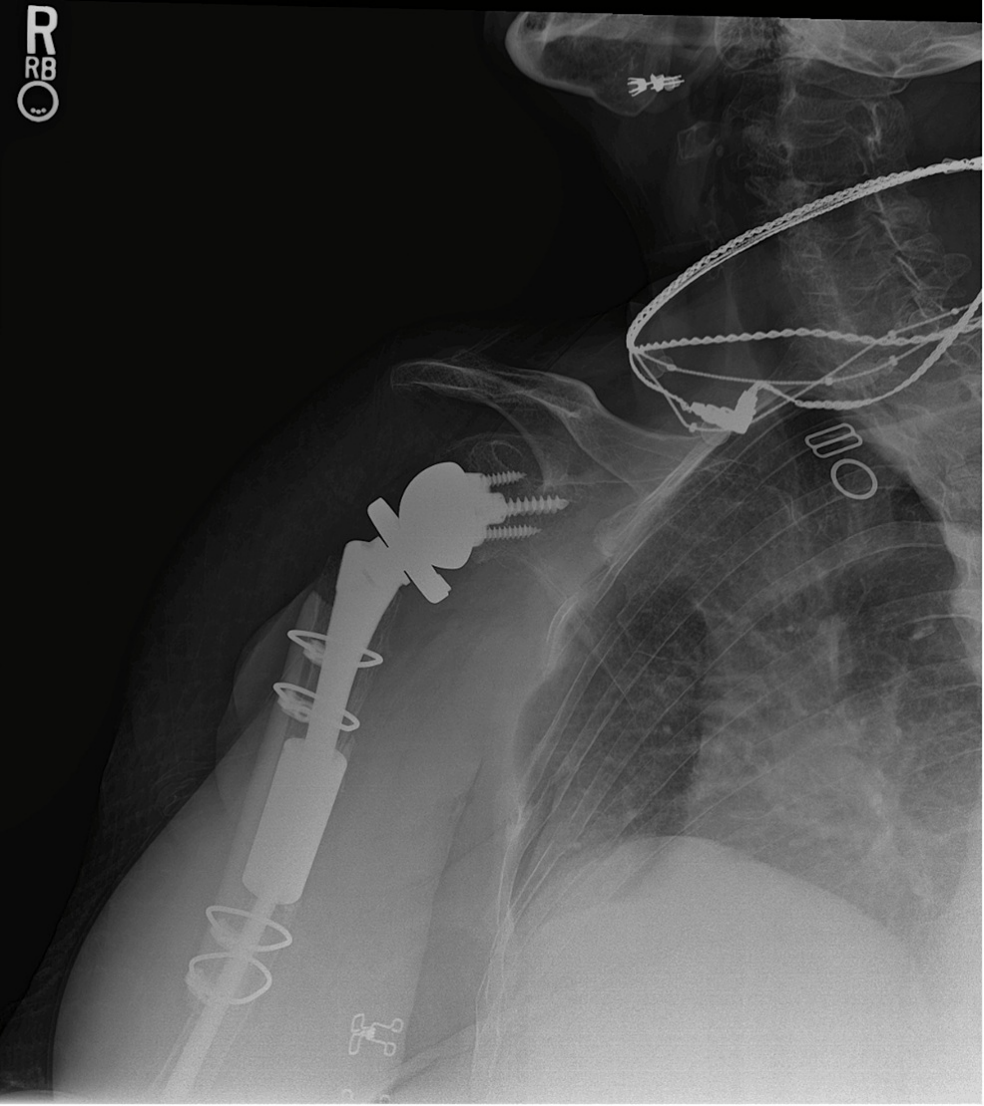
AP radiograph of the right shoulder at six-month follow-up. AP: anteroposterior

**Figure 5:**
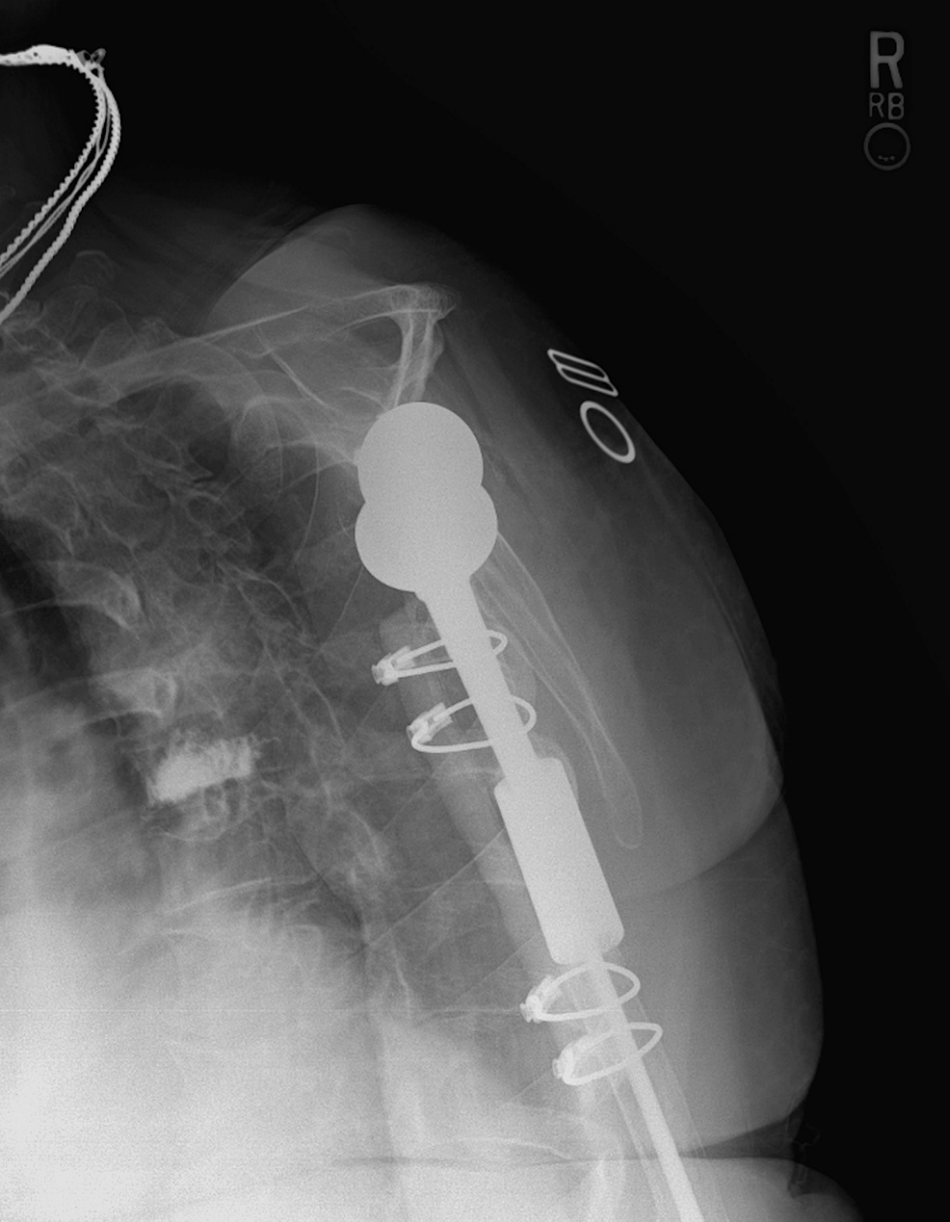
Lateral radiograph of the right shoulder at six-month follow-up.

**Figure 6:**
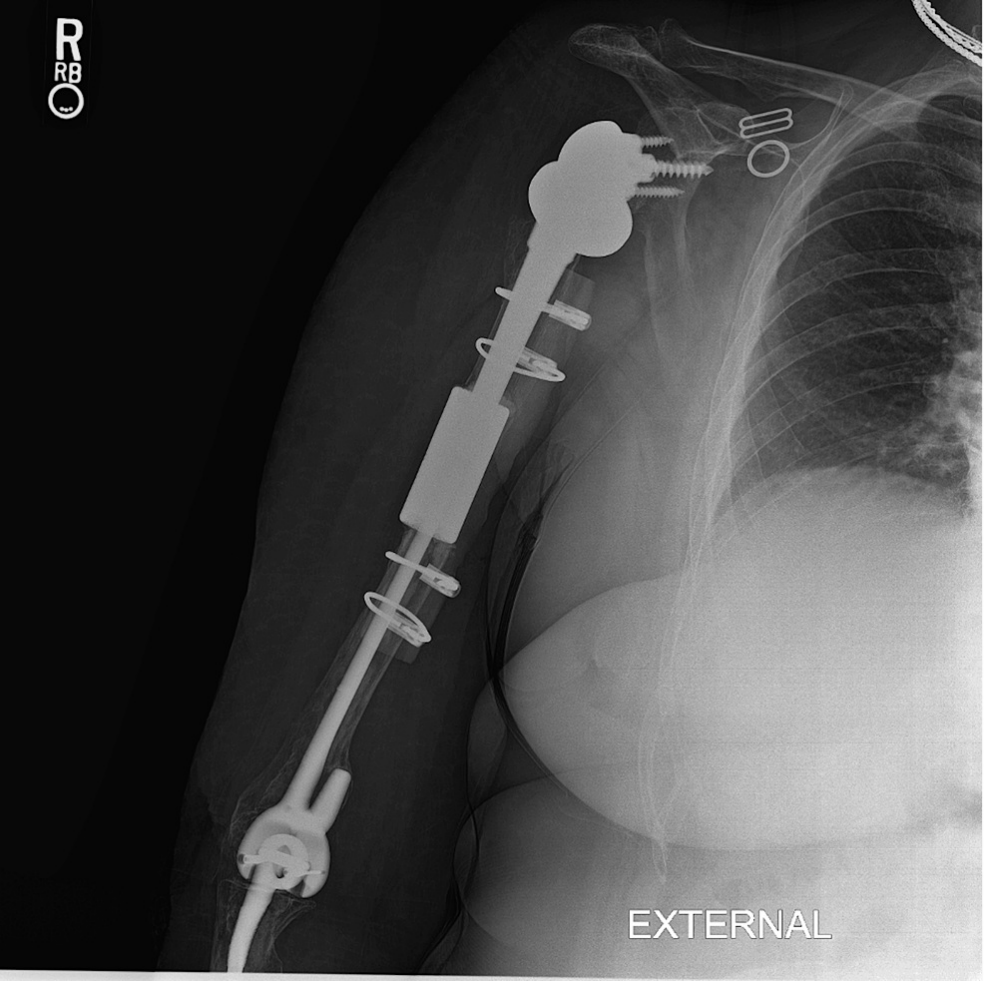
External rotation radiograph of the right humerus at six-month follow-up.

**Figure 7:**
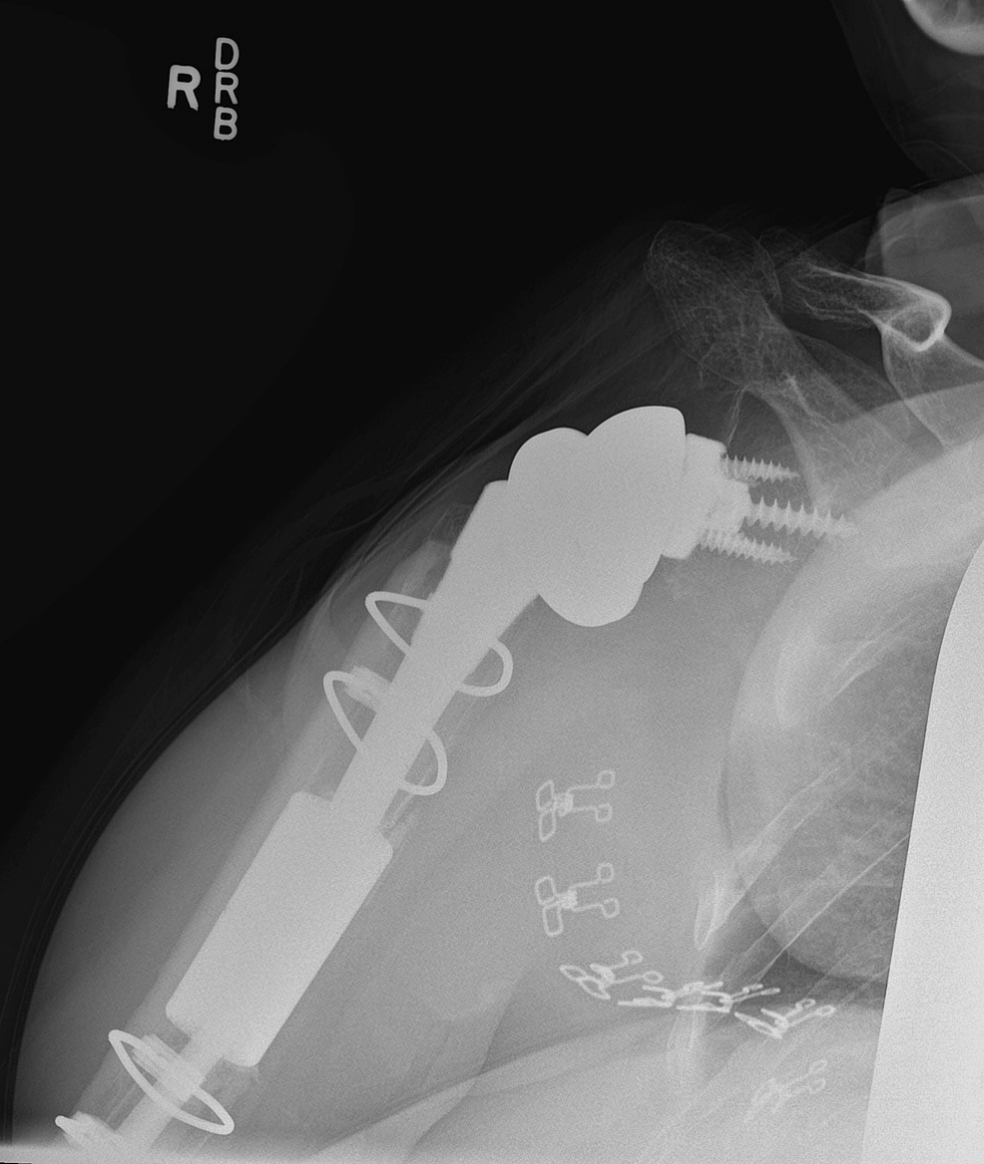
AP oblique radiograph of the right shoulder at 12-month follow-up. AP: anteroposterior

**Figure 8:**
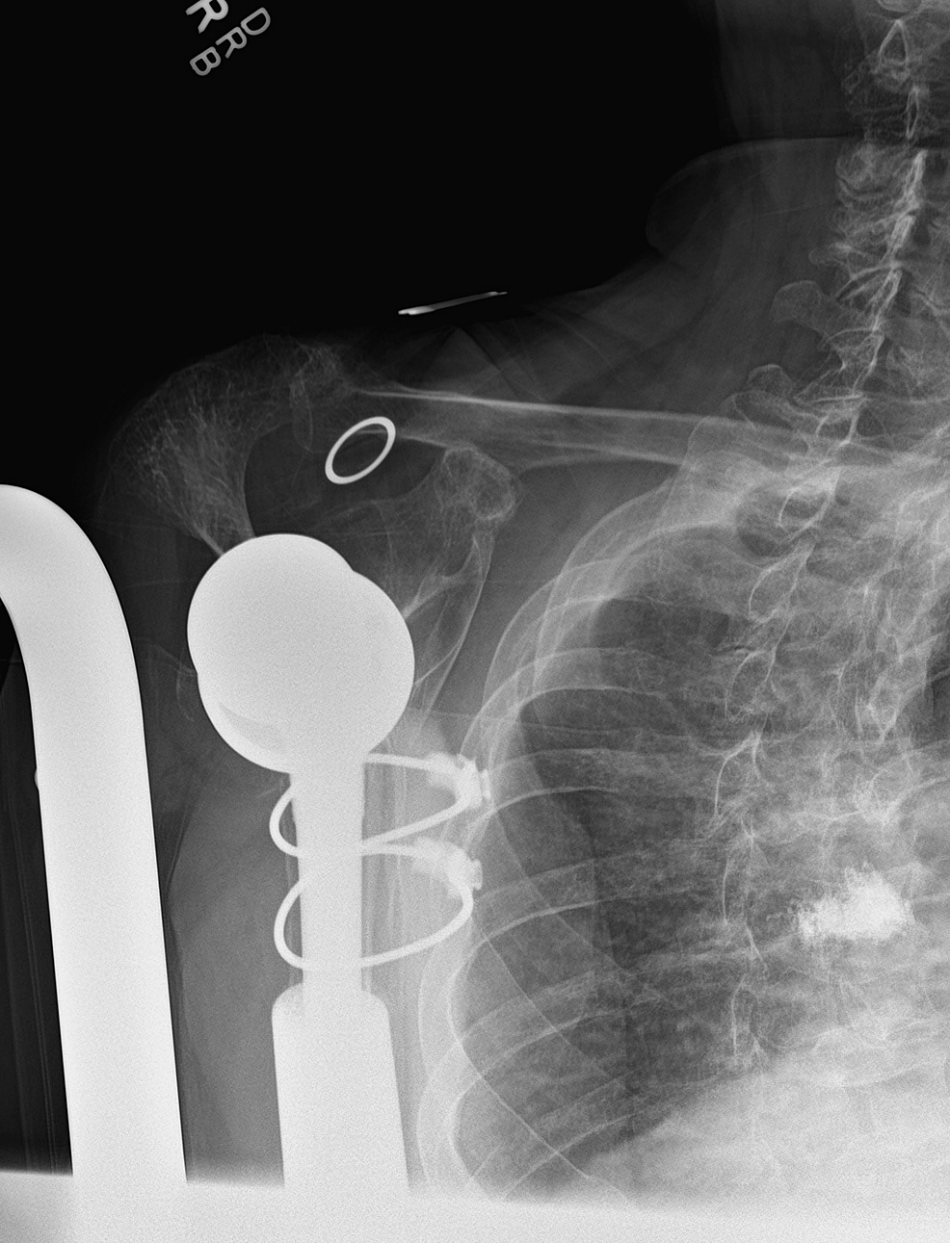
Lateral radiograph of the right shoulder at 12-month follow-up.

**Figure 9:**
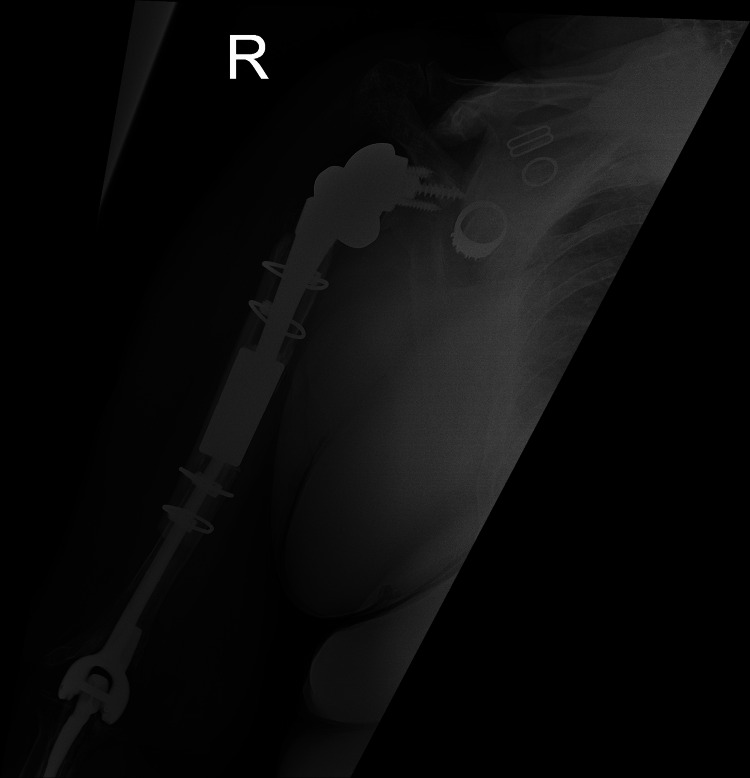
External rotation radiograph of the right humerus at 12-month follow-up.

The patient was instructed to follow up after 10-14 days of strict non-weight-bearing of the right upper extremity with activity as tolerated. At the six-month follow-up appointment, she reported decreased pain and increased functional range of motion. On exam, strength was appreciated in the deltoid muscle, and the shoulder range of motion was increased to 45 degrees flexion and 45 degrees abduction with no gross instability present. Imaging studies indicated all implants were in appropriate alignment as seen in Figure 4, Figure 5, and Figure 6. No evidence of loosening or new fracture was appreciated. At one-year follow-up, the patient reported similar functionality and no new concerns. Imaging revealed a stable construct at this time, as seen in Figure 7, Figure 8, and Figure 9.

## Discussion

Historically, salvage surgery has been implemented as a strategy to avoid amputation and remove large tumor or infection-derived defects while optimizing the remaining structure and functionality of the affected body part [3-5]. Salvage surgery using large-scale allografts is less commonly used to treat fractures, though some data supports its use for complex cases involving the lower extremities [8]. Laumonerie et al. reported a case series that supports the use of an allograft-prosthetic composite in the revision of a failed TEA secondary to aseptic loosening [9]. Despite this study, there is a paucity of literature on upper extremity salvage surgery using allograft for periprosthetic fractures. To our knowledge, this is the first report of RTSA and allograft-composite reconstruction as a combined treatment for a periprosthetic nonunion fracture in the upper extremity. Although Monument et al. noted patients are likely to require additional revision procedures [2], we are hopeful that this patient will continue to have an optimal recovery, as she has for the first 12 months postoperatively. Given the favorable outcomes of this procedure, these findings may support the compounded use of an allograft-composite reconstruction linked to a reverse total shoulder implant as a salvage technique for periprosthetic fractures under conditions such as multiple adjacent implants, bone deformity, and severe osteoarthritis.

## Conclusions

Previous literature suggests upper extremity salvage surgery using large-scale allografts is successful in treating large tumor or infection-derived defects, though data is lacking as to whether this treatment is effective in periprosthetic fractures in patients with significant comorbidities. This patient's success in the postoperative year supports the use of allograft-composite reconstruction followed by linkage to a reverse shoulder implant as a salvage treatment for periprosthetic fractures under certain conditions, such as multiple adjacent implants, bone deformity, and severe osteoarthritis.
